# A Consideration of Dental Patents

**Published:** 1890-07

**Authors:** L. D. Wood

**Affiliations:** Grand Rapids, Mich.


### A Consideration of Dental Patents.

BY L. D. WOOD, D.D.S., GRAND RAPIDS, MICH.

I have very reluctantly consented to undertake the preparation of a paper on this subject, though I am free to confess what will appear later, that it is one in which I never have had any special interest, and of which I have but trifling knowledge. I am not ingorant of the fact that much has been said and written with reference to patents on inventions which find employment in our chosen field of labor, and in a much more acceptable manner than I am able to command; but I am influenced by a desire to discuss it on its merits, without partiality, and by provoking friendly discussion gain more light on a vexed question. I shall, therefore, take the liberty of begging your patient indulgence for the little time I shall occupy in presenting a very imperfect and incomplete view of the subject before us.

Patents, in a general way, are the product of a comparatively recent civilization, dating back only to the time of Luther—the morning star of the Reformation. This has been a period characterized by the most wonderful discoveries and inventions, great progress in the sciences and arts, and a general uplifting of the race to higher standards, nobler actions, and grand achievements. The real, substantial advancement of the world has been made during this time.

It is but little more than two hundred years since modern science began to have any realization of the momentous problems which confronted it and sought their solution, and it was then that legislative enactments were suggested and undertaken looking to the stimulation to the production of inventions and processes, and affording a self-earned reward to their originators. As inventions began slowly to be put forth inventors came to realize the need of some protection for the capital they had invested in their infant industries, and a remedy was found in governmental grants, rights, or as now known, patents.

No savage people ever had a patent law for the reason that their wants are so simple that they have no need for inventions, and consequently no inventors to protect. It therefore follows that patents are not only the outcome of a civilized state of society, but rather belong to it in its most advanced and enlightened conditions, following in the wake of investigation, discovery, learning, development.

A patent may be defined to be an exclusive right granted to an individual to manufacture and sell an article of commerce of his own invention. This right is secured to him for a term of years and becomes an article of property which is transferrable and may be defended in courts of law.

But the question which chiefly concerns us at this time is, "May a dentist or other professional man take out letters-patent on any invention he may produce, having immediate relation to the uses of the profession of which he is a member?" In other words, "Is it professional or the correct thing to do to receive revenue from any thing he may control which by its employment in his specialty will help to lessen the ills and add to the comfort of the human race?" "Ought not all discoveries and inventions become the property of the public if they are adapted to relieve suffering?"

While no question is or has been raised as to the undoubted right of a mechanic patenting the results of his skill and study, shall the man whose occupation is known as a "profession," enjoy the same privilege?

If time allowed it might be profitable to enter into an inquiry as to just what is comprehended in the terms "trade" and "profession." Where shall the dividing line be drawn? And is dentistry a trade or a profession, or both?

If, unfortunately for most writers on this theme, it should be decided that dentistry is chiefly a trade, though of a high order of excellence, the favorite term "professional patents" would become unmeaning and ridiculous for surely it will not be disputed that the occupation of a clergyman or a teacher or an attorney is a professional occupation, and consists in the impartation of ideas, but no one ever heard of ideas being patentable.

If you stand at your velvet-upholstered chair and excavate cavities ever so thoroughly, filling them ever so beautifully, you are doing nothing more or less than mechanical work, and so is the sombre-visaged gentleman who excavates a two-by-six hole in the earth, refilling it with all that is mortal of the once proud professional man. The construction of an artificial denture is no more a professional proceeding than is the manufacture of an artificial limb, building a house, or running a locomotive. It is true there is a difference in the amount or degree of manipulative skill employed, but there need necessarily be no difference in the mental attainments of the operators described.

Now, as I do not wish to cause any unrest in the minds of my confreres as to whether they are engaged in a trade or profession, I will not give the dictionary definition of either. It might rudely disturb the sweet equanimity of some who are always ready to jealously defend every thing mistakenly called professional.

Since dentistry occupies the unfortunate position which it does—that of a connecting link between the trades and professions—and we have seen that it calls for the employment of methods, instruments and appliances which are necessary in a tradesman's pursuits, and are not essentially a part of a strictly professional calling, it would seem that it is the field *par excellence* for the introduction into, and exercise of the highest inventive skill to be had, so as to remove its practices to a plane so far above and beyond mere handicraft and the common usages of trades as to command the respect and esteem to which an unquestioned profession is entitled. In proportion as man becomes enlightened and lifted up in the scale of civilization his wants increase, and to meet and supply those wants the combined efforts of the skillful and inventive in every land are constantly taxed to the utmost extent.

The practitioner who undertakes to meet the demands of modern dentistry, confidently placing his dependence in his own unaided resources, and ignoring the advantages derivable from the inventions of others in his specialty, need not be greatly surprised if his efforts result in an inglorious failure. I hope none will understand me as contending that dentistry is not a profession because its practice calls for the employment of instruments and tools. Nothing of the kind has been intended. Its duties are performed under ever varying conditions, circumstances and surroundings, and its ideas are, or should be, unselfish, expansive, humanizing, ennobling, having to do with the mental, moral, and physical well-being of man. It seeks to alleviate suffering, to extend the average of human existence, and add something to the general fund of knowledge. The man, whether popularly known as a tradesman or professional, who can take a set of natural teeth, covered with calcarous deposits, having devitalized pulps and abscesses, and by treatment and filling restore them to comfort and usefulness, need not be ashamed of his appellation or ability, and if he also possesses the skill and ingenuity to devise an instrument or appliance which shall greatly assist him and his brethren in the duties of their calling, securing patent protection therefor, he is not only a blessing to his profession but a good man of business, and is entitled to praise and encouragement. I have no respect for the nonsense which speaks of a dentist's remuneration for service as

"an accident," nor can I conceive of any good reason why a professional man of any kind should be a failure as a financier. But, alas! too often such is the case. Numerous examples will be recalled of men high in our profession, and fertile in useful but unpatented inventions who have dropped by the wayside, early in the journey of life leaving families in a homeless and penniless condition to mourn their improvidence and want of business enterprise. Charity is a lovely and commendable thing, but by all means let it begin at home. The "dear public" may do for politicians to rave about but those of one's own household should be far dearer to honest men. Some writers talk as though it was little less than criminal to demand a fee or accept of royalties by inventors, but I have yet to meet the man, engaged in professional work, who is laboring altogether for the good of his patrons and from a love of the work without expectation of monetary equivalent. There may be such gentlemen in the legal profession but there are no such dentists.

The reproach from which our calling is a great sufferer is *not* due to the fact that some few of its members have availed themselves of patent protection on some little invention more or less useful, but because there are in our ranks so many charlatans, ignoramuses and quacks, who are successful in deluding the public into the belief that they, and they only, are the men of great learning, nobility of character, and phenomenal skill. Their inventions generally are infringements, their startling discoveries usually turn out to be something resurrected from oblivion. They labor under the supposition that there is no difference between reputation and character, and in the place of knowledge substitute bluster and sarcasm if not falsehood. They advertise to charge a certain small fee but the operation, when performed, always presented unexpected difficulties requiring unparalleled skill, and of course, a large fee is exacted.

These are the men and methods which have done our profession an irreparable injury and brought disgrace to our brethren who have made inventions and patented them in the honorable desire to profit by the labor and skill devoted to their production.

We claim to be a liberal and progressive profession; if we really are and would prove our claim we ought to be very generous in the encouragement we give to the origination and production of appliances which are adapted to use in our field of labor. The tendency of the times is toward greater liberality of thought, tolerance of opinion, practicality of ideas. Let our actions show that we are *en rapport* with this spirit of progression. A high condition of society is only attainable by the more highly diversified employments, liberal education, and elevating moral surroundings and the general culture of society, like that of the individuals composing it, can be reached only by the widest encouragement. Mere human nature has no standard or supreme conscience by which to square or correct itself. So long as this is the case it must be expected that men will widely differ in their deductions and conclusions.

The conservative wing in the present controversy on the patentability of inventions that are used in our specialty claim that patents restrict the use of inventions and humanity suffers in consequence, and they declare that the public weal is to be considered as of greater importance than the rights of the individual inventor. This rule applies in the case of calamities, nuisances, public improvements, etc., but not in the case of inventions. The public demands and is willing to pay for the best obtainable, though it has no technical knowledge of the advantages of one method over another It is the bounden duty of the dentist to analyze and discriminate, making use of the one best suited to the case in hand. If he does not provide himself with the necessary instruments, whether patented or not, to do the work entrusted to his care, in a skillful manner, he is recreant to the confidence reposed in him, and a disgrace to his calling. It can not be denied that there is a commercial side to this question and one whose importance is not to be lightly ignored, though certain fanatical members of the conservative party intimate it is beneath serious consideration. It is little less than infatuation to talk of "having such a consideration for the good of others as to prefer financial loss that they may be benefitted," and to contrast the "evil nature" of the commercial side to the "heavenly spirit" of the professional. Such statements are so manifestly untenable as to be ridiculous.

The fact that an invention is patented is no proof that the consumer has to pay an advanced price for it. Let inventors be deprived of the privilege of securing patents and their efforts will cease to be put forth, as inventors, like other mortals, are not working for their health or the fun of it. I do not believe that the small royalties usually received by patentees cut any figure in raising the selling price of dental commodities. Manufacturers having large capital, improved machinery, and skilled workmen, can produce and sell instruments, or any thing else, much more cheaply than could the individual inventor; hence, it would seem that the present arrangement is the wisest yet known. The proposition has been made that the government, through a commission of qualified examiners, grant inventors bounties commensurate with the importance of their inventions and that the patent system be done away with.

I believe the remedy for many of the evils of which we complain lies within our own power. We have too many dental colleges; their terms are too short and their courses are not thorough enough. Applicants for admission should be subjected to a rigid examination that when once admitted it should denote that they were worthy of respect for their mental attainments.

The possession of a diploma should amount to a guarantee that its holder was truly learned in his profession, and it should be something of an assurance that he was of high moral character, pure motives and noble ambitions.

We have many noble, progressive men, but the *morale* of the profession is not one that will bear too close inspection. There are scores of alleged dentists practicing illegally in this State, but no one seems to interfere with them.

There are members of this society yet living who can recall exhibitions of patented manufactures in its own room by men known to employ some questionable methods, and some of these men have been members of this society.

Gentlemen, these things ought not so to be. The education of our patients is all right and a most commendable thing, but it is not enough; the work should begin in our own hearts and minds. There has been great progress made toward a high ideal in the past generation but there is much more to make. Let us try to lead the public to a higher appreciation of faithful services, striving meanwhile to correct our own failings and shortcomings, and by exercising honesty, integrity, and uprightness we shall be rewarded in the not distant future by seeing dentistry occupying the position of honor to which its usefulness and worth entitle it.

I have purposely taken a middle ground in the preparation of this paper.

---

After the reading of this paper the association adjourned to 9 A.M., Wednesday.

WEDNESDAY MORNING SESSION.

Meeting called to order at 10 A.M. by President Case. Minutes of previous meeting read and approved. On motion of Dr. Harroun all visiting members from outside of the State were allowed the privilege of the floor in discussions.

Dr. Dorrance spoke on Dr. Wood's paper, especially on the point as to who shall be admitted as students to dental colleges and as to how they shall be admitted. As a rule now all dental students enter the college directly without ever having had an office pupilage with a preceptor. A great many come directly to the college without ever having had a moment's conversation with a dentist on the subject of dentistry. Others come on the recommendation of a dentist. Consequently many of the students that enter college are totally unfit to become good practical workmen because they have no mechanical ability to start with, and can not be educated in the limited time at the disposal of the instructors, because of other important duties devolving upon them. Then again some have not sufficient literary attainments to enable them to master the scientific studies in a satisfactory manner. Would suggest some form of office pupilage that would determine whether a man was fit to become a dental student before he could apply for admission to a college.

Dr. T. W. Brophy, of Chicago. We have nineteen chartered dental colleges in Illinois. It is an embarrassing problem with us to have to face the fact that any man or set of men can for three dollars procure from the secretary of our State a license or charter to organize a dental college. But all these colleges in Chicago are not organized to educate dentists; they are organized to make money directly, being run as infirmaries where work is done at cheap rates; or they are organized to advertise the men who conduct them. Every State can support one well conducted dental school, but nineteen are too many for even Illinois. And the above explanation will be sufficient reason why we have so many; they are not all educational institutions.

Colleges are criticised for not making better dentists, but I say the colleges are doing all they can to convert the raw material that comes into their hands into skilful and cultured dentists. Men of all sorts are to be found applying for admission to our colleges. Men with good habits of study, but no mechanical ability, not even a germ that can be cultivated. And then again men of natural mechanical skill, but bad students, or indifferent and lazy men will be found in all colleges. These are some of the things that can not be determined by any preliminary examination. It takes sometimes nearly an entire term to find out that some members of the class will never be a credit to themselves or the school that graduates them. It is my experience that these are the most difficult of all men to convince of their inability. They will protest against the verdict that there is no dentist in them, and insist on being allowed to continue their course; they will refuse to be dismissed or to receive the offer of fees returned, or plead to be allowed to try again to redeem themselves.

Dr. A. M. Long, of Monroe. Would you have the dentist select the student in preference to the college faculty?

Dr. Brophy. I certainly would for this reason. The dentist living in the same community will have better facilities for knowing the habits of a student, whether he is morally fit to enter the profession, whether he has had sufficient educational advantages, and whether he has natural qualifications that will enable him to prepare himself as a skilful workman. A recommendation from a responsible dentist would be a great help to college faculties, and be a means of raising the quality of the students as nothing else can do.

Dr. E. J. Waye, Sandusky, O. Are the dental students of today as thoroughly educated in the scientific branches of the profession as they ought to be?

Dr. Brophy. Yes, the dental student of today is better educated in the medical branches than ever before. Because of the rapid growth of dental art, it is almost impracticable to thoroughly qualify a man in the brief time of a college course.

Dr. Dorrance. If students are to enter the college without an office pupilage, I think they should be put on probation the first year and care should be exercised to determine whether a man is calculated to become a good dentist. If he is found to have no capacity for either the work or study required to fit him to assume the important place of a practitioner, he should be advised to give up the study of dentistry and seek some occupation more congenial to his natural endowments. No preliminary examination, however rigid, could accomplish so much as some such plan as this.

Dr. J. N. Crouse, of Chicago. I think it is absurd for colleges to attempt to shift this matter upon the dentists. One half of the students never consult a dentist about entering the profession, but go directly to the college on their own responsibility, and the responsibility of admitting them rests entirely on the faculties of the colleges. The colleges seem to be entirely satisfied if they find that a man can read and write. This is not enough. Every man should be examined thoroughly, not only as to his literary attainments, but it should be the aim to find out whether he has the qualifications necessary to make a good dentist or not. If not, send him away, and if a man is once admitted and it is afterwards found that a mistake has been made, send him home. The whole trouble in this matter is that every college is striving to get the largest class, and every man that applies and pays his money is received. This is the truth of the matter and the colleges must bear the blame for the incompetent men they are turning into the profession today.

Dr. W. H. Jackson. I believe the fault lies with the examiners of the college. It is a very difficult position and it requires a high degree of moral courage to be able to say no to a man who has set his mind and heart on entering a profession, to say nothing of the difficulties of determining the character of every applicant. And even after they are admitted and found to be lacking in many elements necessary for the making of a good practitioner, it takes considerable courage to inform a man he is not capable of continuing his course of study to the best advantage to himself and will probably make a failure as a practical workman.

Dr. Waye. We should not look at this matter from the selfish standpoint. We owe something to the community upon whom we inflict a poorly prepared man. An inferior dentist is capable of inflicting incalculable injury upon the unsuspecting public, and a man with the diploma of a reputable dental college to give him a standing can do more harm and be sustained in it, than a man that every one recognizes as a tyro.

Dr. Brophy. When we receive a man into college and take his fee, we are bound to give him a course of instruction, and it is perfectly absurd to expect that any preliminary examination will determine what a man may be or become after he has been through a course of systematic training and study in a dental college. No dental faculty has the time or wisdom to do all that has been suggested in any large measure of cases.

Dr. Crouse. It is the duty of your faculties to take the time to sift out the worthless men if it takes a year to do it. You can't shift the responsibility onto the dental profession, we won't have it.

Dr. A. J. Swasey, of Chicago. Dr. Brophy has told us that we have a large number of dental colleges in Chicago, but he hasn't, according to my notion, given the true reason for this condition of things. The fact is, there are a great many wealthy dentists in Chicago and they adopt this plan for disposing of their wealth. He didn't know whether Dr. Brophy would reject a man any sooner who hadn't a hundred dollars than one who had, but he was of the opinion that some sort of a preliminary course of study either in the college or in the form of an indenture with a preceptor was desirable. This indenture to be considered as a part of the course, and if, at its termination, the student was considered qualified to enter upon a regular college course of study, he should be entered and given proper credit for the time spent with the preceptor. In all cases a fee should be paid for this preliminary course.

Dr. Metcalf said that it was evident from the discussion that some colleges are run for the money there is in them, and that if a student could read and write and had the necessary fee, he could enter almost any college he liked. But he knew there were colleges that were not run on this basis, and he thought it was the duty of the profession to select the college to which students should be sent. We should send our students to those colleges in which we know there is no opportunity for the faculty to admit unworthy men to their classes for the sake of the fee they bring. He was glad to say that we had such an institution in our own State to which this objection would not apply.

Dr. Wood was very much gratified that so much interesting discussion had been called out on this point, but he would like to hear something in regard to dentists who are illegally practicing in this State.

Dr. G. E. Corbin, of St. Johns. I have been secretary of the State Examining Board for several years and I have a great many letters notifying me of persons practicing illegally. If I can judge from these letters there are more persons now practicing illegally than ever before. The trouble is that no one is willing to prosecute these persons. A person does not like to do this work even if he should feel it to be his duty to do so. I have heard from one man four times in a little over a year in as many different parts of the State. On motion, subject passed.

Being favorably reported by the Board of Censors, the following persons were elected members of the association: W. L. Williams, M. D. Vanderberg, F. H. Essig, L. P. Hall, E. L. Dillman, L. C. Smith, —— Furgeson, H. C. Raymond.

The secretary read a circular letter that had been sent out as an advertising circular by Dr. L. L. Davis, of Detroit, in which he used a letter from one of the secretaries of the dental section of the Ninth International Medical Congress, asking him to give a clinic in operative dentistry at the meeting in Washington, to call attention to superior ability as an operative dentist.

On motion the matter was referred to the Board of Directors for action.

Dr. G. E. Corbin read a paper entitled,

